# Prediction of anticancer peptides derived from the true lectins of *Phoenix dactylifera* and their synergetic effect with mitotane

**DOI:** 10.3389/fphar.2024.1322865

**Published:** 2024-02-23

**Authors:** Othman Baothman, Ehab M. M. Ali, Salman Hosawi, Emadeldin Hassan E. Konozy, Isam M. Abu Zeid, Abrar Ahmad, Hisham N. Altayb

**Affiliations:** ^1^ Department of Biochemistry, Faculty of Science, King Abdulaziz University, Jeddah, Saudi Arabia; ^2^ Center of Artificial Intelligence in Precision Medicines, King Abdulaziz University, Jeddah, Saudi; ^3^ Division of Biochemistry, Chemistry Department, Faculty of Science Tanta University, Tanta, Egypt; ^4^ Laboratory of Proteomics and Glycoproteins, Biotechnology Park, Africa City of Technology, Khartoum, Sudan; ^5^ Pharmaceutical Research and Development Centre, Faculty of Pharmacy, Karary University, Omdurman, Sudan; ^6^ Department of Biological Sciences, Faculty of Science, King Abdulaziz University, Jeddah, Saudi Arabia

**Keywords:** date palm, MCF-7 breast cancer cells, HepG2, liver cancer, *in silico* analysis

## Abstract

**Background and aims:** Cancer continues to be a significant source of both illness and death on a global scale, traditional medicinal plants continue to serve as a fundamental resource of natural bioactive compounds as an alternative source of remedies. Although there have been numerous studies on the therapeutic role of *Phoenix dactylifera*, the study of the role of peptides has not been thoroughly investigated. This study aimed to investigate the anticancer activity of lectin peptides from *P. dactylifera* using *in silico* and *in vivo* analysis.

**Methods:** Different computational tools were used to extract and predict anticancer peptides from the true lectins of *P. dactylifera*. Nine peptides that are bioactive substances have been investigated for their anticancer activity against MCF-7 and T47D (two forms of breast cancer). To counteract the unfavorable effects of mitotane, the most potent peptides (U3 and U7) were combined with it and assessed for anticancer activity against MCF-7 and HepG2.

**Results:** *In silico* analysis revealed that nine peptides were predicted with anticancer activity. In cell lines, the lowest IC_50_ values were measured in U3 and U7 against MCF-7 and T47D cells. U3 or U7 in combination with mitotane demonstrated the lowest IC_50_ against MCF-7 and HepG2. The maximum level of cell proliferation inhibition was 22% when U3 (500 µg/mL) and 25 µg/mL mitotane were combined, compared to 41% when 25 µg/mL mitotane was used alone. When mitotane and U3 or U7 were combined, it was shown that these bioactive substances worked synergistically with mitotane to lessen its negative effects. The combination of peptides and mitotane could be regarded as an efficient chemotherapeutic medication having these bioactive properties for treating a variety of tumors while enhancing the reduction of side effects.

## Introduction

Over the past 2 decades, there has been a lot of interest in investigating natural materials for bioactive compounds as an alternative source of remedy (Alvin et al., 2014). Traditional medicine is a form of healthcare that draws upon the accumulated wisdom, techniques, and abilities rooted in the theories, customs, and historical insights specific to diverse societies. It is employed to maintain wellness and to address, diagnose, enhance, or remedy both physical and mental health conditions (Wangkheirakpam, 2018). Globally, traditional medicinal plants continue to serve as a fundamental resource for primary healthcare, a fact particularly evident in rural areas where people heavily depend on them (Wangkheirakpam, 2018). An approximate figure of 50,000–70,000 medicinal plants are utilized globally (Wachtel-Galor and Benzie, 2011). The World Health Organization (WHO) has formulated a strategy for traditional medicine aimed at integrating it into the official policies of national healthcare systems (Organization, 2001). This strategy highlights the significance of guaranteeing the safety, efficacy, and quality of medicinal plants. It also emphasizes the need to improve their availability and affordability while promoting their responsible and rational use in therapy (Hassan et al., 2020). In Saudi Arabia (KSA), the traditional utilization of ethnomedicinal plants reflects a deep connection between customary remedies, health, dietary habits, and traditional healing traditions that are unique to particular cultures (Aati et al., 2019).

Lifestyle diseases are of growing concern. Many prevalent diseases including type-2 diabetes, cardiovascular diseases, obesity, and cancer are reducing life span (Li et al., 2020). Plant-based natural compounds have a long history of providing preventative therapeutic benefits against these diseases (Najmi et al., 2022). Nowadays most of the research become oriented toward finding new alternative natural source products, a derivative of natural origin, or even semi-synthetic drugs based on natural product models from plants and animals to replace pure synthetic pharmaceutical drugs and hence, reduce their negative side effects (Pan et al., 2013).

The plant natural products fall into two categories: primary metabolites such as proteins and carbohydrates and secondary metabolites such as alkaloids, flavonoids, steroids, saponin, glycosides, tannin, carotenoid, and phenolic components (Silva et al., 1998). The primary metabolites such as some proteins and peptides provide an arsenal of defense molecules, including lectins, ribosome-inactivating proteins, protease inhibitors, antimicrobial proteins and peptides (AMP-ACPs), ureases, etc (Kocyigit et al., 2023).

The AMP are highly abundant in higher plants especially Caryophyllaceae and Rhamnaceae families (Abdoullahi et al., 2022). Such peptides were reported to have potent antitumor activities which are cherimolacyclopeptide C, cycloheptapeptide, dianthins E, etc. The other type of AMPs is the antimicrobial proteins which consist of around 12 families of which three members are reported to have antitumor activities (Abdoullahi et al., 2022). Because of the antitumor activities of these proteins and peptides, they can be also named ACPs (anticancer proteins/peptides).

*Phoenix dactylifera L* (the date palm tree) holds the distinction of being among the most ancient and fundamental crops in the Middle East, Southwest Asia, and North Africa (Al-Alawi et al., 2017). The scientific name of the plant is *P. dactylifera L.* cv. *Hillaw* and the scientific classification is Kingdom: Plantae. Phylum: Magnoliophyta. Class: Liliopsida. Subclass: Arecidae. Order: Arecales. Family: Arecaceae. Genus: *Phoenix*. Species: *dactylifera* (Al Sawafi and Al Maliki, 2020).

Lectins are a varied category of proteins from different families that possess the ability to recognize carbohydrates (Osman et al., 2023). Lectins are proteins that can bind to mono- or oligosaccharides in a specific and reversible manner, and they are characterized by having at least one non-catalytic domain for this purpose (Mishra et al., 2019). These proteins are found in all organisms from all kingdoms and play several roles including immunological and defense functions. It has been proven that some lectins trigger apoptosis and/or death of tested cancer cells (Rabelo et al., 2012). Thousands of lectins have indeed been isolated from all kinds of organisms, yet the search for new ones especially from plant sources is still a frontier area of study (Srikanth and Chen, 2016). Figuring out the way to solve their immunogenicity, increase their stability, identify their mode of action as defense proteins and engineer lectins for drug use and/or drug delivery systems are persistent issues scientists are trying to tackle (Dhuna et al., 2005).

Breast cancer stands as the most prevalent form of cancer across the globe and is the primary cause of cancer-related mortality among women (Organization, 2023). According to a recent WHO report, around the globe there are more than 2.3 million cases of breast cancer in both genders are reported annually (Organization, 2023).

In this study, we aim to study the anticancer activity (targeting breast cancer and liver cancer cells) of peptides derived from the true lectin of *P. dactylifera*, using *in silico* and *in vivo* analysis.

## Methodology

### Lectins putative genes from *Phoenix dactylifera* with ACPs motifs

A genome-wide search of *P. dactylifera* for lectin families and putative domains was done using NCBI nucleotide and protein databases, more details are found in our previous publication (Osman et al., 2023).

### Screening of putative true lectins peptides

Eight true lectins were identified in *P. dactylifera* and they were legume (XP_038973335.1, XP_008793992.2), Malectin (XP_008795773.2, XP_008812544.3, XP_038974428.1, XP_008792302.2 and XP_026655880.2) and M-Type (XP_038985107.1). The above-mentioned sequences were screened for the presence of putative peptides using the PeptideCutter server, and three proteolytic gastric enzymes (Pepsin (pH1.3), Trypsin and Chymotrypsin) were used with the default setting.

### *In silico* prediction of anticancer activity and toxicity of identified peptides

Different tools were used to screen the identified peptides for possible anticancer activity. AntiCP2.0 web tool (Agrawal et al., 2021) and Peptide Calculator were used to predict peptides with anticancer activity, hydrophobicity, Hydrophilicity, Hydropathicity, and charge. The CellPPD (Gautam et al., 2013) was used to predict cell penetration activity and other physical properties. CancerPPD database (Tyagi et al., 2015) was used for aligning peptide sequences with anticancer peptides. ToxinPred was used to screen toxic/non-toxic peptides (Gupta et al., 2013).

### Peptide synthesis and *in vitro* anticancer activity

According to *in silico* analysis 9 peptides (U1, U2, U3, U4, U5, U6, U7, U8, and U9) (Peptide lable) were selected due their predicted anticancer activity and lower toxicity. These peptides were synthetized commercially by GenScript Biotech Corporation (GenScript, Piscataway, NJ) and they were dissolved in ultrapure water in different concentrations ranged from 10 µM −250 µM.

MCF-7, T47D (breast cancer), and HepG2 (liver cancer) were donated by the Tissue Culture Unit, Department of Biochemistry, Faculty of Science, King Abdulaziz University. Selected human cell lines were cultured in DMEM media with 10% fetal bovine serum (FBS) at 37°C in a CO_2_ incubator. After 70%–90% confluence, 5 mL of 0.25% trypsin is injected to separate the cells. The cells were counted using trypan blue, and the cell concentration was set at 10^5^/mL. A 96-well plate was filled with 100 mL in each well, and the plate was then incubated for 24 h.

Mitotane, marketed as Lysodren, functions as a steroidogenesis inhibitor and cytostatic antineoplastic drug. It is employed for treating adrenocortical carcinoma and Cushing’s syndrome (Lo Iacono et al., 2021). In this study the media in each well was replaced to contain media containing peptides in varying concentrations (U1, U2, U3, U4, U5, U6, U7, U8, and U9). U3 and U7, which have the lowest IC_50_ values, were chosen to be combined with mitotane. Mitotane concentrations ranged from 20 to 600 µM. U3 and U7 were combined with mitotane using two different preparation techniques. Various concentrations of U3 or U7 (40–1500 µM) and mitotane (20–600 µM) were serially diluted. serial dilution of mitotane concentration with 500 µg/mL of U3 or U7 at a fixed concentration Each concentration was repeated four times.

After 48 h of incubation at 37°C, 100 µL of the 0.5 mg/mL MTT was changed with media containing the medication. At 37°C in the dark, the 96-well plate was incubated for 4 h. After removing the MTT and replacing it with 100 µL of DMSO, the plate was allowed to rest for 15 min. The absorbance of each well in 96 well plate was read at wave length 595 nm by ELISA reader (Bio-RAD microplate reader, Japan).

### 2.7 Statistical analysis

The treated cells viability and IC_50_ were expressed as the mean ± standard deviation (SD). The percentage viability was calculated by multiplying the absorbance of treated cells by 100 and dividing the absorbance of untreated cells. The software GraphPad Prism (version 9.0, San Diego, CA, United States) was conducted to calculate the IC_50_ of peptides, mitotane and their combination.

## Results and discussion

### *In silico* analysis

The genome-wide search of *P. dactylifera* yielded 11 lectin families, of which five families had sequences with ACP motifs (28 seq. total). Only eight sequences are reported to be true lectins (containing all amino acids required for sugar-binding) (Supplementary Table S1). Usually, ACPs are short sequence peptides their length ranging from of 10–50 aa (Chen et al., 2021). Accordingly, the digestion of true lectins by proteolytic gastric enzymes (Pepsin (pH1.3), Trypsin and Chymotrypsin) revealed the presence 96 putative peptides ranging in length from 9 to 20 aa as shown in Table 1, most of these peptides were non-toxic (Supplementary Table S2). AntiCP2.0 web tool revealed that 9 of these peptides were predicted with anticancer probabilities that are all scored more than 0.60 (Supplementary Table S3), suggesting their ACPs probability (Liu et al., 2022). The charge of four peptide (U1, U3, U6 and U8) was positive or zero, which increase their chance to interact with negatively charged cancer cells (Liu et al., 2022). Additionally, the Grand Average of Hydropathy Value for protein sequences (GRAVY) was recorded using by using Peptide Calculator tool. The GRAVY value represents the sum of hydropathy values of peptide residues divided by the protein length, where positive values indicate hydrophobic and negative values indicate hydrophilic (Avraamides et al., 2021). Two peptides (U3 and U6) scored 0.29 and 0.81 kcal/mol and were hydrophobic while the rest were hydrophilic. Previous studies have uncovered that ACPs possess a significant degree of hydrophobicity and a positive overall charge (Table 2). This enables them to selectively target and destroy cancer cells by interacting with the negatively charged components of cancer cell membranes (Huang et al., 2021).

**TABLE 1:** True lectins peptide sequences.

Sequence ID	Lectin families	Peptide sequence	Peptide mass [Da]	Peptide length [aa]
XP_038973335.1	Legume	MVYPEPVTIYDEASVIN	1940.195	17
		SSYPSAIPDL	1049.145	10
		TLPSTVVAVE	1015.172	10
		DISANHIGIDVHTIYSVVQ	2081.313	19
		SSQGYGSSPSPTPR	1407.459	14
		EYISEVTIISR	1309.482	11
		HEGWEQCVIHR	1393.543	11
		AGTMGYIAPEYAITGK	1642.887	16
		DTPLPALPPNMPIPMF	1751.132	16
		NVPAPPPNSESCSIV	1510.682	15
XP_008793992.2	Legume	NQADSSPSS	891.847	9
		LSPYPSEIPENSYGGT	1710.814	16
		NVEYNDSSSNHVGIDVHTIF	2247.362	20
		DACVGYDGGAK	1055.127	11
		HWSTSYVVD	1093.161	9
		ASVVGAGVVA	828.964	10
		EQVEMAVAIDS	1191.319	11
		EYISEVTIISR	1309.482	11
		HEEWEQCVVHR	1451.579	11
		VDHDCDPATTV	1172.232	11
		DAPLPILSPNMPLPVF	1721.088	16
		LRPPPVDISN	1107.275	10
XP_008795773.2	Malectin	NGSIPATWASL	1116.239	11
		IDGNPISGK	899.999	9
		IDMQGTSMEGPFPPTF	1754.990	16
		LAPSSCQEGN	1005.067	10
		NMVSSYSSTESNSIAR	1732.840	16
		DDHEYEDDPSQMGPSR	1877.870	16
		IFDVSIQGQK	1134.297	10
		EANGTGRPIIK	1155.319	11
		LISAISVTPNF	1161.363	11
		TDSKPDIQESK	1247.324	11
		FINEIGMISA	1094.291	10
		YGCCIEGSQ	959.056	9
		IYEYMENNS	1162.236	9
		NEEENTHISTR	1329.346	11
		IAGTMGYMAPEYA	1374.589	13
		VNTSVNIDQSSK	1291.381	12
		NSSSSNISHQAV	1230.256	12
XP_008795773.2	Malectin	SVDPCSGNAGW	1092	11
		IDGNPISGK	900	9
		LAPSSCQEGN	1005	10
		SSTESNSIAR	1051	10
		EDDPSQMGPSR	1218	11
		EANGTGRPIIK	1155	11
		LISAISVTPNF	1161	11
		TDSKPDIQESK	1247	11
		INEIGMISA	947.1	9
		NEEENTHISTR	1329	11
		EVISGMSNTNY	1214	11
		VNTSVNIDQSSK	1291	12
XP_038974428.1	Malectin	SVDPCSGNAGW	1092	11
		IDGNPISGK	900	9
		LAPSSCQEGN	1005	10
		SSTESNSIAR	1051	10
		EDDPSQMGPSR	1218	11
		EANGTGRPIIK	1155	11
		LISAISVTPNF	1161	11
		TDSKPDIQESK	1247	11
		INEIGMISA	947.1	9
		NEEENTHISTR	1329	11
		EVISGMSNTNY	1214	11
		TSVNIDQSSK	1078	10
		NSSSSNISHQAV	1230	12
XP_008792302.2	Malectin	SVDPCSGDAAW	1107	11
		IDGNPITGK	914	9
		DMQGTSMEGPFPSI	1497	14
		TESPPANCW	1004	9
		SSTNINSIASC	1096	11
		INCGGSHVTVDGNEY	1565	15
		EDDTSPQGASR	1162	11
		LISAISVTPNF	1161	11
		GIVAASCVVIM	1062	11
		PDGSEIAVK	915	9
		INEIGMISA	947.1	9
		DEEENTHISTR	1330	11
XP_026655880.2	Malectin	AVGSASPTL	801.9	9
		GVDPCSGEGNW	1120	11
		LESDVVCDCS	1069	10
		EGPIPSGISN	970	10
		NCSIHGDIPAY	1189	11
		TVGSSGITQC	952	10
		EGSVNTVECY	1100	10
		TFPCSASNK	954.1	9
		HINCGGEETIIK	1313	12
		MDDDVNADNY	1171	10
		NIEDAAGGPGKPVIK	1466	15
		LISAISVTPNF	1161	11
		DVGSPSSNR	917.9	9
		TIIVVVVMA	944.2	9
XP_038985107.1	M-Type	HDNTAHPAPDS	1161	11
		GGSDGGPGGGNK	958.9	12
		VNDIIIKPNDR	1296	11
		HNLLCPETVES	1241	11
		DDVTAMVPR	1003	9
		NTEAHSFPV	1001	9

**TABLE 2:** Peptide properties.

Peptide ID	Peptide sequence	Charge	Hydrophobicity	GRAVY	Hydrophilicity
U1	SSQGYGSSPSPTPR	1	0.14	−1.4	−0.27
U2	NQADSSPSS	−1	0.46	−1.5	−0.32
U3	NGSIPATWASL	0.0	−0.69	0.29	0.08
U4	NEEENTHISTR	−1.5	0.87	−2.08	−0.47
U5	DEEENTHISTR	−2.5	1.13	−2.08	−0.48
U6	AVGSASPTL	0	−0.46	0.81	0.11
U7	NCSIHGDIPAY	−0.5	−0.4	−0.09	−0.01
U8	TFPCSASNK	1	−0.07	−0.47	−0.18
U9	HDNTAHPAPDS	−1	0.37	−1.64	−0.27

### Experimental procedure

**FIGURE 1:**
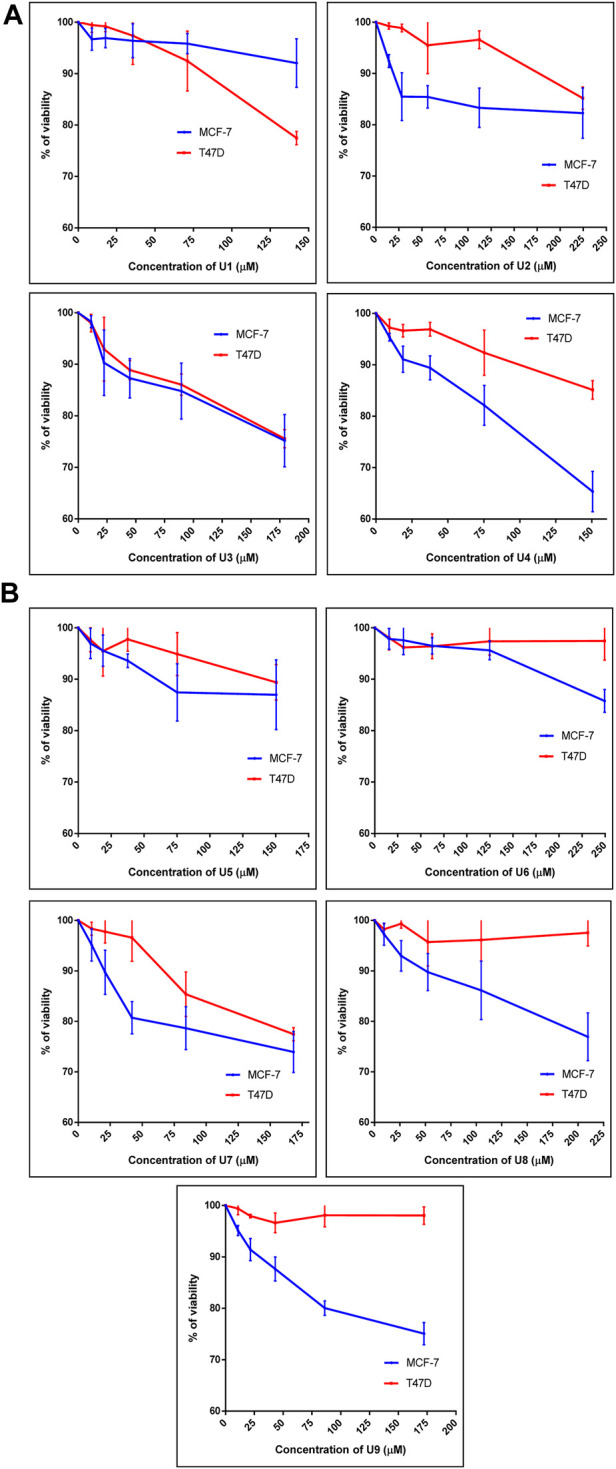
Percentage of cell viability of MCF-7 and T47D treated with peptide U1, U2, U3, U4 **(A)** and U5, U6, U7, U8, U9 **(B)**. Cells treated with peptides were incubated for 48 h and cell viability was evaluated using an MTT assay. Cell viabiltity percentage were obtained by the logarithm of the concentration of each peptide. The IC_50_ value was determined using GraphPad. Experiments were performed three times independently (*n* = 3).

The percentage of viability of both human breast cancer cell lines (MCF-7 and T47D) treated with 9 bioactive peptides is shown in Figure 1. Nine bioactive peptides with varying quantities were found in *P. dactylifera* true lectins after being exposed to MCF-7 and T47D for 48 h. These nine bioactive peptides’ IC_50_ values were calculated in two different types of breast cancer cells (Table 3). The lowest IC_50_ values measured in U3 and U7 against MCF-7 were 480 and 348 µM, respectively, whereas 517 and 584 µM were recorded against T47D. The table below shows the greatest IC_50_ values for U1, U5, and U6 against MCF-7 and T47D, demonstrating reduced efficacy on both breast cell types. U2, U4, U8, and U9 each had an IC_50_ of 681, 297, 640, and 417 µM against MCF-7, whereas their IC50s against T47D ranged from 871 to 5462 µM, indicating that their effects on T47D were attenuated (Table 3). Interestingly, Wang et al. (Wang et al., 2019) identified an alternatively-activated pathway for both metastatic breast and liver cancers involving the enrichment of cytokine-cytokine receptor in both cases. This finding could support our results, where peptides exhibited activity against both cancer types, suggesting the potential for targeting common pathways associated with these cancers.

**TABLE 3:** IC_50_ of peptides (µM (U1, U2, U3, U4 U5, U6, U7, U8,U9) against MCF-7 and T47D.

Group (A)	U1	U2	U3	U4	
MCF-7	1007–2258	451.2–1030	373.4–617.4	260.6–338.5	
Range	1507 ± 71.70	681.8 ± 32.03	480.1 ± 55.04	297 ± 19.72	
Value					
T47D	457.6–784.5	1141–1988	434.7–616.5	722.7–1051	
Range value	599.1 ± 20.37	1506 ± 78.03	517.6 ± 34.86	871.5 ± 23.37	
Group (B)	U5	U6	U7	U8	U9
MCF-7	548.7–1089	1359–2075	265.9–457.8	518.2–792.5	351.3–496.3
Range	773.1 ± 44.46	1680 ± 74.31	348.9 ± 25.32	640.8 ± 43.82	417.5 ± 15.69
Value					
T47D	866.1–1829	2522–13781	485.3–702.8	1993 to 11,143	2615–11410
Range value	1259 ± 62.47	5896 ± 118.59	584 ± 29.99	4713 ± 83.59	5462 ± 99.29

### Synergetic effect mitotane

Mitotane accumulates cholesterol lipoproteins in the adrenal cortex and adipose tissues that is used for the biosynthesis of corticosteroid hormones. Mitotane causes the cytochrome P450 enzymes to be out of control and the mitochondrial membranes to become depolarized, so mitotane damages the adrenal cortex. Mitotane is used for the treatment of advanced adrenocortical cancer as well as postoperative adjuvant therapy. Zona fasciculate and reticularis of the adrenal cortex are particularly affected by the high dose of mitotane-caused neuro-cellular toxicity (Lo Iacono et al., 2021).

Mitotane is used in combination with the peptides U3 and U7 to increase its therapeutic effects while lowering its side effects. Higher doses of mitotane have more commonly been linked to central neurological damage (Kasperlik-Zaluska, 2000; Fassnacht et al., 2018).

**FIGURE 2:**
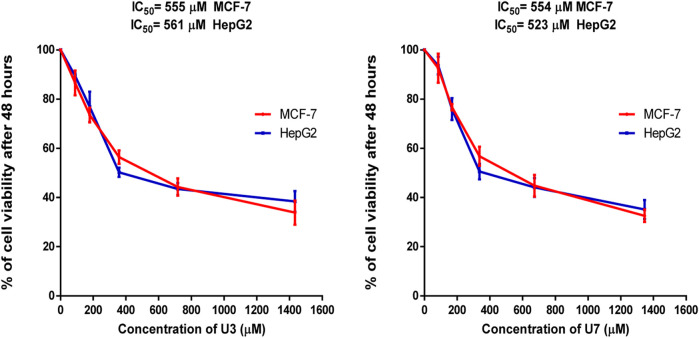
Percentage of viability of MCF-7 and HepG2 treated with U3 and U7. Cells treated with U3 and U7 peptides were incubated for 48 h and cell viability was evaluated using an MTT assay. Cell viabiltity percentage were obtained by the logarithm of the concentration of each peptide. The IC_50_ value was determined using GraphPad. Experiments were performed three times independently (*n* = 3).

In this investigation, the percentage of viability of MCF-7 and HepG2 treated with various concentrations ranging from 40–1500 µM U3 and U7 was evaluated to determine the IC_50_ of U3 and U7 against both cells (Figure 2). The IC_50_ values of U3 and U7 ranged from 523 to 561 µM against MCF-7 and HepG2 and were both high and almost identical (Figure 2; Table 4).

**TABLE 4:** IC_50_ (µM) of combined U3 and U7 peptides with mitotane against MCF-7 and HepG2.

	U3	U7	Mitotane	Mitotane + U3*	Mitotane + U7*
MCF-7	486.6–634.2	480.9–639.5	62.41–93.33	45.60–59.63	44.00–55.04
Range	555.5 ± 43.46	554.5 ± 44.35	76.32 ± 5.04	52.14 ± 5.51	49.21 ± 1.99
Value					
HepG2	454.4–692.5	427.0–642.5	32.44–49.69	20.85–26.78	29.66–43.68
Range value	561 ± 39.27	523.8 ± 35.82	40.15 ± 4.69	23.63 ± 1.87	36 ± 1.61
	Mitotane + U3	Mitotane + U7			
	500^#^	500^#^			
MCF-7	27.37–35.71	40.31–51.72			
Range	31.27 ± 2.85	45.66 ± 5.48			
Value					
HepG2	43.56–75.36	33.77–53.40			
Range value	57.29 ± 1.65	42.46 ± 1.46			

*The serial dilution of concentration of mitotane ranging from 10, to 250 µM) and various concentrations of U3 or U7 (40–1500 µM/mL).

^#^Serial dilution of mitotane concentration with a fixed concentration of U3 or U7 (500 µg/mL).

The IC_50_ values for mitotane against HepG2 and MCF-7 cells were 31.27 and 57.29 µM HepG2 has shown greater anticancer activity efficacy than MCF-7. The HepG2 IC_50_ is 2-fold lower than MCF-7 (Table 4). The combination of U3 and U7 with mitotane with different concentrations of mitotane and peptides (U3 or U7) of HepG2 has nearly the same anticancer activity as cells treated alone with mitotane, although this combination is only marginally effective against MCF-7.

**FIGURE 3:**
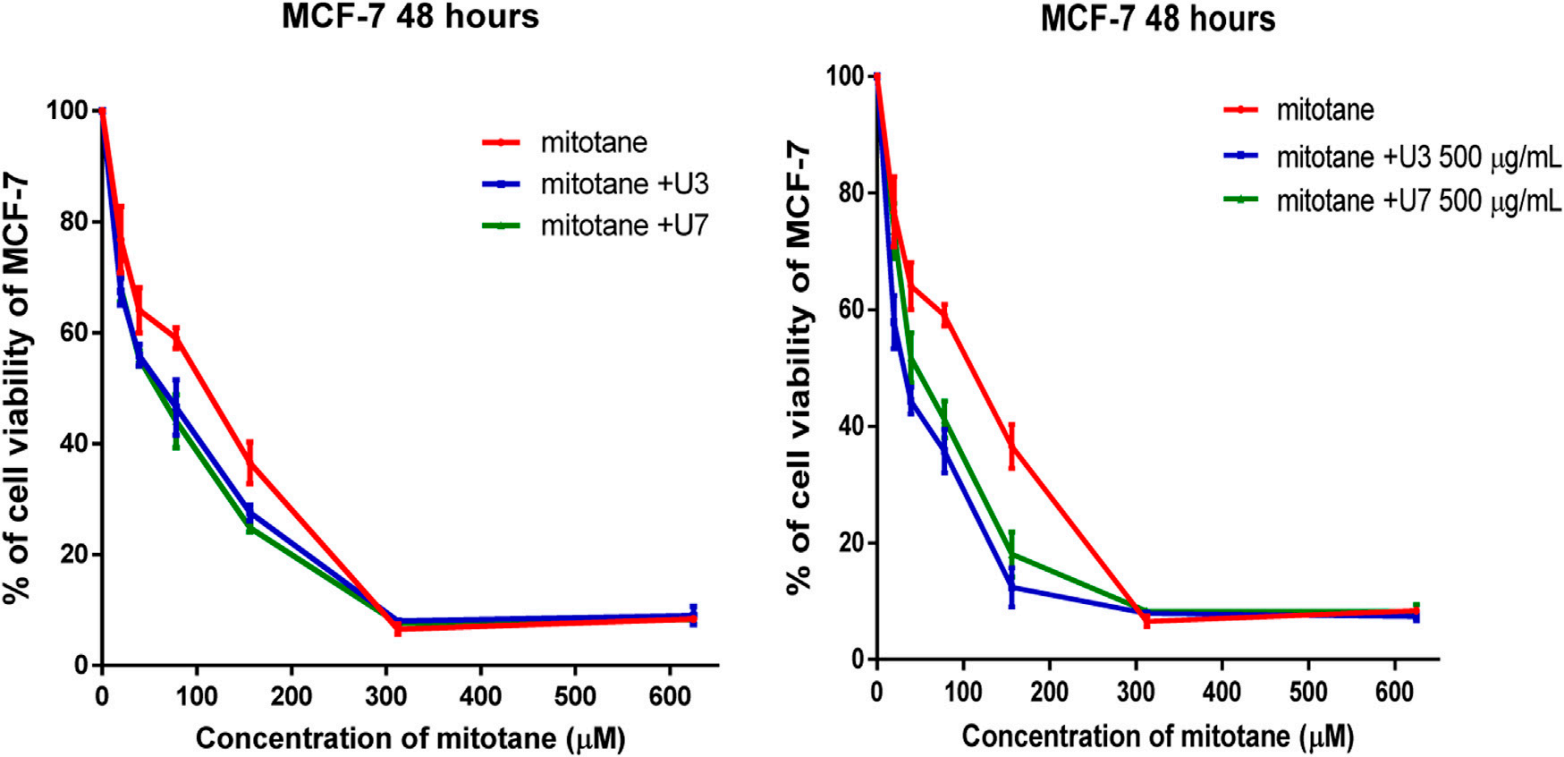
Percentage of viability of MCF-7 treated with mitotane combined with U3 and U7 The serial dilution of concentration of mitotane (20–600 µM) and various concentrations of U3 or U7 (40–1500 µM). Serial dilution of mitotane concentration with a fixed concentration of U3 or U7 (500 µg/mL). Cells treated with mitoten, peptides and their combinations were incubated for 48 h and cell viability was evaluated using an MTT assay. Cell viabiltity percentage were obtained by the logarithm of the concentration of each peptide. The IC_50_ value was determined using GraphPad. Experiments were performed three times independently (*n* = 3).

**FIGURE 4:**
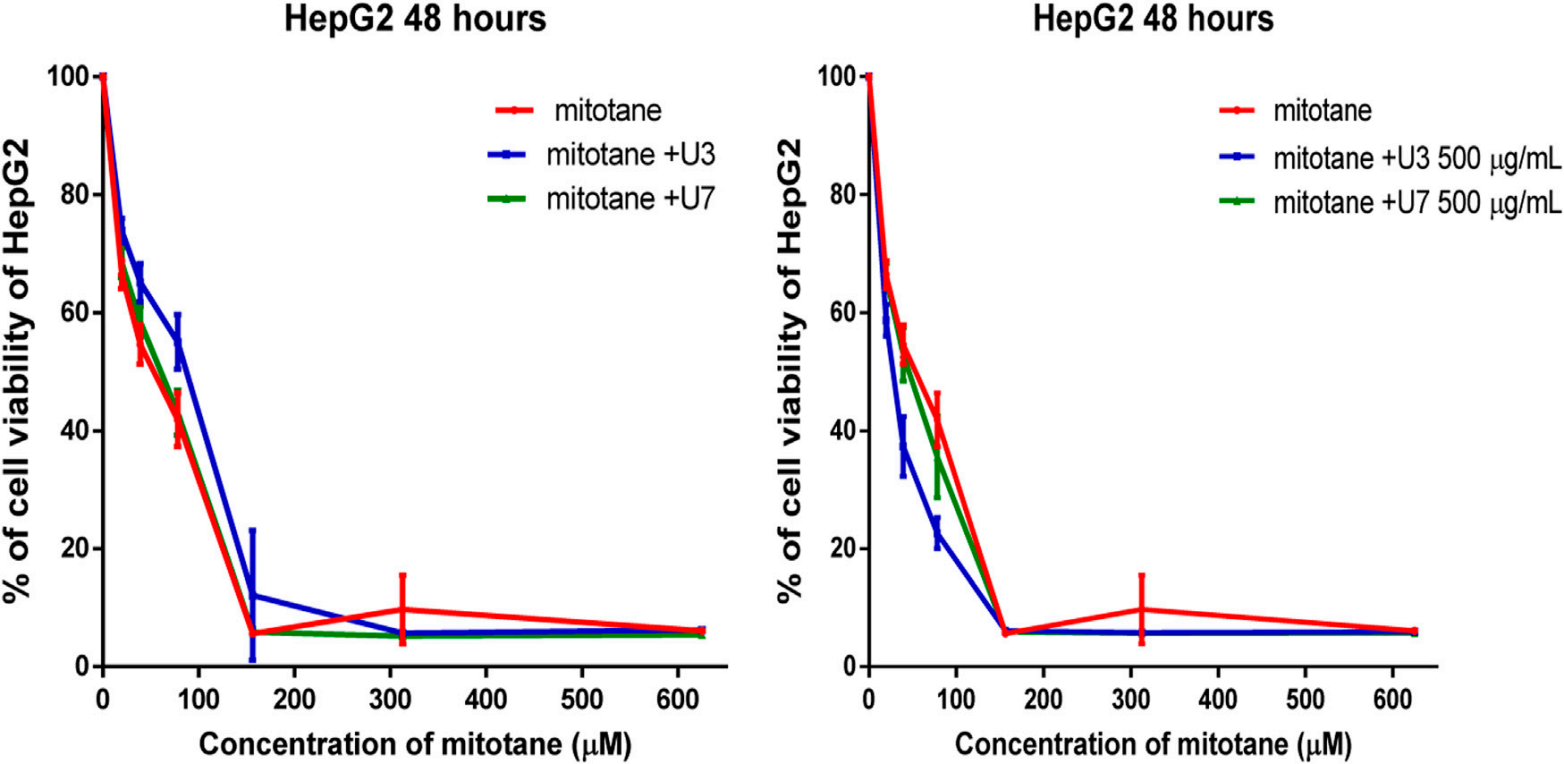
Percentage of viability of HepG2 treated with mitotane combined with U3 and U7. The serial dilution of concentration of mitotane (20–600 µM) and various concentrations of U3 or U7 (40–1500 µM). Serial dilution of mitotane concentration with a fixed concentration of U3 or U7 (500 µg/mL). Cells treated with mitoten, peptides and their combinations were incubated for 48 h and cell viability was evaluated using an MTT assay. Cell viabiltity percentage were obtained by the logarithm of the concentration of each peptide. The IC_50_ value was determined using GraphPad. Experiments were performed three times independently (*n* = 3).

It is more effective to combine various mitotane doses with a constant amount of U3 or U7 (500 µg/mL), which is effective against both MCF-7 and HepG2. In contrast, the combination of mitotane with U3 (500 µg/mL) was more effective against MCF-7 and HepG2, with an IC50 that was 2.4 and 1.7 times lower than cells treated with mitotane alone. The combination of mitotane and U3 (500 µg/mL) is more effective than the combination of mitotane and U7 Table 4 and Figures 3, 4.

**FIGURE 5:**
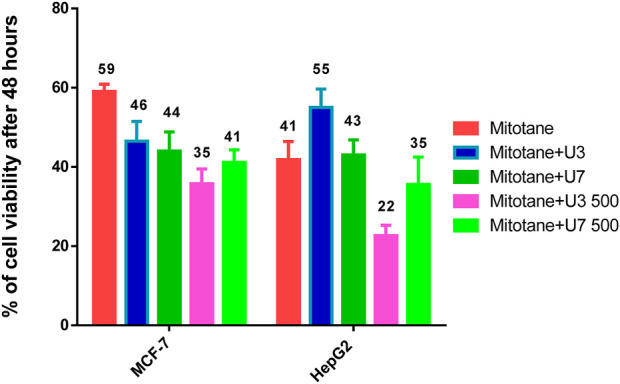
% of viability of MCF-7 and HepG2 treated with combined mitotane at conc 25 µg/mL. The data obtained from Figures 3, 4.

HepG2 cells were treated with mitotane (25 µg/mL) alone, in combination with U3 or U7 (200 µg/mL), and the viability percentages were 41.86 ± 4.54, 55 ± 4.64, and 43 ± 3.78, respectively. The anticancer activity of mitotane (25 µg/mL (80 µM) alone and in combination with U3 (22.62 ± 2.63) or U7 (35.68 ± 6.92) with concentration (500 µg/mL) was more strongly influenced by the percentage of inhibition HepG2 treated with these drugs. The viability percentages of MCF-7 treated with mitotane (25 µg/mL) alone, mixed with U3 or U7 with concentration (200 µg/mL were, respectively, 59.05 ± 1.83, 46.53 ± 4.94, and 44.04 ± 4.77. The percentage of MCF-7 cells that were treated with mitotane (25 µg/mL) alone or in combination with U3 (35.76 ± 3.74) or U7 (41.19 ± 3.16) with concentration (500 µg/mL) had a greater impact on the effectiveness of the anticancer treatment (Figure 5).

## Conclusion

The most bioactive compounds in the current study were U3 and U7, which were successful in treating liver cancer (HepG2) and breast cancer (MCF-7 and T47D). U3 and U7 have high IC_50_, hence mitotane is utilized in combination with these. Compared to cells treated with mitotane alone, the IC_50_ of mitotane and U3 against MCF-7 and HepG2 were reduced to 2.4 and 1.7 times, respectively. Negative mitotane effects may be less likely due to the synergistic effects of mitotane with U3 and U7. It is advised to understand the mechanism by which U3 or U7 combined with mitotane inhibits cell proliferation. Consideration should be given to chemotherapeutic medications that have been combined with these bioactive molecules as a novel way to improve therapy effectiveness and lessen side effects.

## Data Availability

The original contributions presented in the study are included in the article/Supplementary Materials, further inquiries can be directed to the corresponding authors.
