# Supplementary material for: Prediction of anticancer peptides derived from the true lectins of Phoenix dactylifera and their synergetic effect with mitotane
Source: Front Pharmacol. 2024 Feb 23;15:1322865. doi: 10.3389/fphar.2024.1322865 (PMC10920327; doi:10.3389/fphar.2024.1322865)
Supplement: Supplementary file 1 [file Table1.DOCX]

Table S1: Phoenix dactylifera Lectin families and sequence IDs

| **Lectin families** | **Sequence ID** | **Functional lectins** |
| --- | --- | --- |
| **GNA** | XP_008775776.2 |  |
|  | XP_038973918.1 |  |
|  | XP_038973919.1 |  |
| **Legume** | XP_038973335.1 | True-lectin |
|  | XP_008793992.2 | True-lectin |
|  | XP_008801603.2 |  |
|  | XP_008777366.2 |  |
|  | XP_008793619.2 |  |
|  | XP_008776549.1 |  |
| **LysM** | XP_008810487.1 |  |
| **Malectin** | XP_038981350.1 |  |
|  | XP_008795773.2 | True-lectin |
|  | XP_038970403.1 |  |
|  | XP_008812544.3 | True-lectin |
|  | XP_038970404.1 |  |
|  | XP_038974966.1 |  |
|  | XP_038974987.1 |  |
|  | XP_038970401.1 |  |
|  | XP_038970402.1 |  |
|  | XP_038974965.1 |  |
|  | XP_038970403.1 |  |
|  | XP_038974986.1 |  |
|  | XP_038974428.1 | True-lectin |
|  | XP_008792302.2 | True-lectin |
|  | XP_026655880.2 | True-lectin |
|  | XP_038970401.1 |  |
| **M-Type** | XP_038985107.1 | True-lectin |

Table S2. Prediction of all peptide toxicity and general properties

| [**Peptide ID**](https://webs.iiitd.edu.in/raghava/toxinpred/multi_submitfreq_S.php?ran=57599) | [**Peptide Sequence**](https://webs.iiitd.edu.in/raghava/toxinpred/multi_submitfreq_S.php?ran=57599) | [**SVM Score**](https://webs.iiitd.edu.in/raghava/toxinpred/multi_submitfreq_S.php?ran=57599) | [**Prediction**](https://webs.iiitd.edu.in/raghava/toxinpred/multi_submitfreq_S.php?ran=57599) | [**Hydrophobicity**](https://webs.iiitd.edu.in/raghava/toxinpred/multi_submitfreq_S.php?ran=57599) | [**Hydropathicity**](https://webs.iiitd.edu.in/raghava/toxinpred/multi_submitfreq_S.php?ran=57599) | [**Hydrophilicity**](https://webs.iiitd.edu.in/raghava/toxinpred/multi_submitfreq_S.php?ran=57599) | [**Charge**](https://webs.iiitd.edu.in/raghava/toxinpred/multi_submitfreq_S.php?ran=57599) | [**Mol wt**](https://webs.iiitd.edu.in/raghava/toxinpred/multi_submitfreq_S.php?ran=57599) |
| --- | --- | --- | --- | --- | --- | --- | --- | --- |
|  | [MVYPEPVTIYDEASVIN](https://webs.iiitd.edu.in/raghava/toxinpred/pepsearch_S.php?seq=MVYPEPVTIYDEASVIN&thval=0.0) | -1.07 | Non-Toxin | 0.03 | 0.24 | -0.32 | -3 | 1940.4 |
|  | [SSYPSAIPDL](https://webs.iiitd.edu.in/raghava/toxinpred/pepsearch_S.php?seq=SSYPSAIPDL&thval=0.0) | -0.34 | Non-Toxin | -0.01 | -0.03 | -0.25 | -1 | 1049.3 |
|  | [TLPSTVVAVE](https://webs.iiitd.edu.in/raghava/toxinpred/pepsearch_S.php?seq=TLPSTVVAVE&thval=0.0) | -1.45 | Non-Toxin | 0.11 | 1.09 | -0.43 | -1 | 1015.3 |
|  | [DISANHIGIDVHTIYSVVQ](https://webs.iiitd.edu.in/raghava/toxinpred/pepsearch_S.php?seq=DISANHIGIDVHTIYSVVQ&thval=0.0) | -1.01 | Non-Toxin | 0.04 | 0.42 | -0.47 | -1 | 2081.6 |
|  | [SSQGYGSSPSPTPR](https://webs.iiitd.edu.in/raghava/toxinpred/pepsearch_S.php?seq=SSQGYGSSPSPTPR&thval=0.0) | -0.75 | Non-Toxin | -0.27 | -1.4 | 0.14 | 1 | 1407.6 |
|  | [EYISEVTIISR](https://webs.iiitd.edu.in/raghava/toxinpred/pepsearch_S.php?seq=EYISEVTIISR&thval=0.0) | -0.83 | Non-Toxin | -0.09 | 0.24 | 0 | -1 | 1309.6 |
|  | [HEGWEQCVIHR](https://webs.iiitd.edu.in/raghava/toxinpred/pepsearch_S.php?seq=HEGWEQCVIHR&thval=0.0) | -0.35 | Non-Toxin | -0.24 | -1.05 | 0.05 | 0 | 1393.7 |
|  | [AGTMGYIAPEYAITGK](https://webs.iiitd.edu.in/raghava/toxinpred/pepsearch_S.php?seq=AGTMGYIAPEYAITGK&thval=0.0) | -0.53 | Non-Toxin | 0.05 | 0.13 | -0.36 | 0 | 1643.1 |
|  | [DTPLPALPPNMPIPMF](https://webs.iiitd.edu.in/raghava/toxinpred/pepsearch_S.php?seq=DTPLPALPPNMPIPMF&thval=0.0) | -0.9 | Non-Toxin | 0.08 | 0.2 | -0.51 | -1 | 1751.4 |
|  | [NVPAPPPNSESCSIV](https://webs.iiitd.edu.in/raghava/toxinpred/pepsearch_S.php?seq=NVPAPPPNSESCSIV&thval=0.0) | -0.13 | Non-Toxin | -0.06 | -0.14 | -0.13 | -1 | 1510.9 |
|  | [NQADSSPSS](https://webs.iiitd.edu.in/raghava/toxinpred/pepsearch_S.php?seq=NQADSSPSS&thval=0.0) | -0.59 | Non-Toxin | -0.32 | -1.5 | 0.46 | -1 | 891.95 |
|  | [LSPYPSEIPENSYGGT](https://webs.iiitd.edu.in/raghava/toxinpred/pepsearch_S.php?seq=LSPYPSEIPENSYGGT&thval=0.0) | -0.49 | Non-Toxin | -0.09 | -0.84 | -0.09 | -2 | 1711 |
|  | [NVEYNDSSSNHVGIDVHTIF](https://webs.iiitd.edu.in/raghava/toxinpred/pepsearch_S.php?seq=NVEYNDSSSNHVGIDVHTIF&thval=0.0) | -0.55 | Non-Toxin | -0.09 | -0.39 | -0.19 | -2 | 2247.7 |
|  | [DACVGYDGGAK](https://webs.iiitd.edu.in/raghava/toxinpred/pepsearch_S.php?seq=DACVGYDGGAK&thval=0.0) | -0.16 | Non-Toxin | -0.09 | -0.28 | 0.29 | -1 | 1055.3 |
|  | [HWSTSYVVD](https://webs.iiitd.edu.in/raghava/toxinpred/pepsearch_S.php?seq=HWSTSYVVD&thval=0.0) | -1.1 | Non-Toxin | -0.04 | -0.31 | -0.67 | -0.5 | 1093.3 |
|  | [ASVVGAGVVA](https://webs.iiitd.edu.in/raghava/toxinpred/pepsearch_S.php?seq=ASVVGAGVVA&thval=0.0) | -0.92 | Non-Toxin | 0.3 | 2.06 | -0.72 | 0 | 829.1 |
|  | [EQVEMAVAIDS](https://webs.iiitd.edu.in/raghava/toxinpred/pepsearch_S.php?seq=EQVEMAVAIDS&thval=0.0) | -1.3 | Non-Toxin | -0.03 | 0.33 | 0.22 | -3 | 1191.5 |
|  | [EYISEVTIISR](https://webs.iiitd.edu.in/raghava/toxinpred/pepsearch_S.php?seq=EYISEVTIISR&thval=0.0) | -0.83 | Non-Toxin | -0.09 | 0.24 | 0 | -1 | 1309.6 |
|  | [HEEWEQCVVHR](https://webs.iiitd.edu.in/raghava/toxinpred/pepsearch_S.php?seq=HEEWEQCVVHR&thval=0.0) | -0.57 | Non-Toxin | -0.33 | -1.35 | 0.35 | -1 | 1451.7 |
|  | [VDHDCDPATTV](https://webs.iiitd.edu.in/raghava/toxinpred/pepsearch_S.php?seq=VDHDCDPATTV&thval=0.0) | -0.22 | Non-Toxin | -0.15 | -0.36 | 0.29 | -2.5 | 1172.4 |
|  | [DAPLPILSPNMPLPVF](https://webs.iiitd.edu.in/raghava/toxinpred/pepsearch_S.php?seq=DAPLPILSPNMPLPVF&thval=0.0) | -1.33 | Non-Toxin | 0.13 | 0.67 | -0.59 | -1 | 1721.3 |
|  | [LRPPPVDISN](https://webs.iiitd.edu.in/raghava/toxinpred/pepsearch_S.php?seq=LRPPPVDISN&thval=0.0) | -0.88 | Non-Toxin | -0.18 | -0.46 | 0.14 | 0 | 1107.4 |
|  | [NGSIPATWASL](https://webs.iiitd.edu.in/raghava/toxinpred/pepsearch_S.php?seq=NGSIPATWASL&thval=0.0) | -1.25 | Non-Toxin | 0.08 | 0.29 | -0.69 | 0 | 1116.4 |
|  | [IDGNPISGK](https://webs.iiitd.edu.in/raghava/toxinpred/pepsearch_S.php?seq=IDGNPISGK&thval=0.0) | -0.87 | Non-Toxin | -0.11 | -0.57 | 0.32 | 0 | 900.13 |
|  | [IDMQGTSMEGPFPPTF](https://webs.iiitd.edu.in/raghava/toxinpred/pepsearch_S.php?seq=IDMQGTSMEGPFPPTF&thval=0.0) | -0.91 | Non-Toxin | 0 | -0.28 | -0.23 | -2 | 1755.2 |
|  | [LAPSSCQEGN](https://webs.iiitd.edu.in/raghava/toxinpred/pepsearch_S.php?seq=LAPSSCQEGN&thval=0.0) | -0.6 | Non-Toxin | -0.16 | -0.6 | 0.07 | -1 | 1005.2 |
|  | [NMVSSYSSTESNSIAR](https://webs.iiitd.edu.in/raghava/toxinpred/pepsearch_S.php?seq=NMVSSYSSTESNSIAR&thval=0.0) | -0.87 | Non-Toxin | -0.23 | -0.59 | 0.02 | 0 | 1733.1 |
|  | [DDHEYEDDPSQMGPSR](https://webs.iiitd.edu.in/raghava/toxinpred/pepsearch_S.php?seq=DDHEYEDDPSQMGPSR&thval=0.0) | -0.65 | Non-Toxin | -0.45 | -2.3 | 1.11 | -4.5 | 1878.1 |
|  | [IFDVSIQGQK](https://webs.iiitd.edu.in/raghava/toxinpred/pepsearch_S.php?seq=IFDVSIQGQK&thval=0.0) | -1.43 | Non-Toxin | -0.07 | 0.04 | -0.09 | 0 | 1134.5 |
|  | [EANGTGRPIIK](https://webs.iiitd.edu.in/raghava/toxinpred/pepsearch_S.php?seq=EANGTGRPIIK&thval=0.0) | -0.61 | Non-Toxin | -0.21 | -0.7 | 0.43 | 1 | 1155.5 |
|  | [LISAISVTPNF](https://webs.iiitd.edu.in/raghava/toxinpred/pepsearch_S.php?seq=LISAISVTPNF&thval=0.0) | -1.42 | Non-Toxin | 0.18 | 1.29 | -0.86 | 0 | 1161.5 |
|  | [TDSKPDIQESK](https://webs.iiitd.edu.in/raghava/toxinpred/pepsearch_S.php?seq=TDSKPDIQESK&thval=0.0) | -0.97 | Non-Toxin | -0.45 | -1.93 | 1.24 | -1 | 1247.5 |
|  | [FINEIGMISA](https://webs.iiitd.edu.in/raghava/toxinpred/pepsearch_S.php?seq=FINEIGMISA&thval=0.0) | -0.88 | Non-Toxin | 0.2 | 1.18 | -0.62 | -1 | 1094.4 |
|  | [YGCCIEGSQ](https://webs.iiitd.edu.in/raghava/toxinpred/pepsearch_S.php?seq=YGCCIEGSQ&thval=0.0) | 1.26 | Toxin | -0.05 | -0.04 | -0.29 | -1 | 959.18 |
|  | [IYEYMENNS](https://webs.iiitd.edu.in/raghava/toxinpred/pepsearch_S.php?seq=IYEYMENNS&thval=0.0) | -1.01 | Non-Toxin | -0.19 | -1.22 | -0.11 | -2 | 1162.4 |
|  | [NEEENTHISTR](https://webs.iiitd.edu.in/raghava/toxinpred/pepsearch_S.php?seq=NEEENTHISTR&thval=0.0) | -0.68 | Non-Toxin | -0.47 | -2.08 | 0.87 | -1.5 | 1329.5 |
|  | [IAGTMGYMAPEYA](https://webs.iiitd.edu.in/raghava/toxinpred/pepsearch_S.php?seq=IAGTMGYMAPEYA&thval=0.0) | -0.6 | Non-Toxin | 0.11 | 0.35 | -0.61 | -1 | 1374.8 |
|  | [VNTSVNIDQSSK](https://webs.iiitd.edu.in/raghava/toxinpred/pepsearch_S.php?seq=VNTSVNIDQSSK&thval=0.0) | -0.81 | Non-Toxin | -0.24 | -0.67 | 0.19 | 0 | 1291.6 |
|  | [NSSSSNISHQAV](https://webs.iiitd.edu.in/raghava/toxinpred/pepsearch_S.php?seq=NSSSSNISHQAV&thval=0.0) | -0.66 | Non-Toxin | -0.18 | -0.6 | -0.18 | 0.5 | 1230.4 |
|  | [SVDPCSGNAGW](https://webs.iiitd.edu.in/raghava/toxinpred/pepsearch_S.php?seq=SVDPCSGNAGW&thval=0.0) | -0.06 | Non-Toxin | -0.04 | -0.31 | -0.24 | -1 | 1092.3 |
|  | [IDGNPISGK](https://webs.iiitd.edu.in/raghava/toxinpred/pepsearch_S.php?seq=IDGNPISGK&thval=0.0) | -0.87 | Non-Toxin | -0.11 | -0.57 | 0.32 | 0 | 900.13 |
|  | [LAPSSCQEGN](https://webs.iiitd.edu.in/raghava/toxinpred/pepsearch_S.php?seq=LAPSSCQEGN&thval=0.0) | -0.6 | Non-Toxin | -0.16 | -0.6 | 0.07 | -1 | 1005.2 |
|  | [SSTESNSIAR](https://webs.iiitd.edu.in/raghava/toxinpred/pepsearch_S.php?seq=SSTESNSIAR&thval=0.0) | -0.94 | Non-Toxin | -0.33 | -0.91 | 0.47 | 0 | 1051.2 |
|  | [EDDPSQMGPSR](https://webs.iiitd.edu.in/raghava/toxinpred/pepsearch_S.php?seq=EDDPSQMGPSR&thval=0.0) | -0.72 | Non-Toxin | -0.43 | -1.98 | 1.05 | -2 | 1218.4 |
|  | [EANGTGRPIIK](https://webs.iiitd.edu.in/raghava/toxinpred/pepsearch_S.php?seq=EANGTGRPIIK&thval=0.0) | -0.61 | Non-Toxin | -0.21 | -0.7 | 0.43 | 1 | 1155.5 |
|  | [LISAISVTPNF](https://webs.iiitd.edu.in/raghava/toxinpred/pepsearch_S.php?seq=LISAISVTPNF&thval=0.0) | -1.42 | Non-Toxin | 0.18 | 1.29 | -0.86 | 0 | 1161.5 |
|  | [TDSKPDIQESK](https://webs.iiitd.edu.in/raghava/toxinpred/pepsearch_S.php?seq=TDSKPDIQESK&thval=0.0) | -0.97 | Non-Toxin | -0.45 | -1.93 | 1.24 | -1 | 1247.5 |
|  | [INEIGMISA](https://webs.iiitd.edu.in/raghava/toxinpred/pepsearch_S.php?seq=INEIGMISA&thval=0.0) | -0.83 | Non-Toxin | 0.15 | 1 | -0.41 | -1 | 947.25 |
|  | [NEEENTHISTR](https://webs.iiitd.edu.in/raghava/toxinpred/pepsearch_S.php?seq=NEEENTHISTR&thval=0.0) | -0.68 | Non-Toxin | -0.47 | -2.08 | 0.87 | -1.5 | 1329.5 |
|  | [EVISGMSNTNY](https://webs.iiitd.edu.in/raghava/toxinpred/pepsearch_S.php?seq=EVISGMSNTNY&thval=0.0) | -0.95 | Non-Toxin | -0.08 | -0.35 | -0.3 | -1 | 1214.5 |
|  | [TSVNIDQSSK](https://webs.iiitd.edu.in/raghava/toxinpred/pepsearch_S.php?seq=TSVNIDQSSK&thval=0.0) | -0.8 | Non-Toxin | -0.28 | -0.88 | 0.36 | 0 | 1078.3 |
|  | [NSSSSNISHQAV](https://webs.iiitd.edu.in/raghava/toxinpred/pepsearch_S.php?seq=NSSSSNISHQAV&thval=0.0) | -0.66 | Non-Toxin | -0.18 | -0.6 | -0.18 | 0.5 | 1230.4 |
|  | [SVDPCSGDAAW](https://webs.iiitd.edu.in/raghava/toxinpred/pepsearch_S.php?seq=SVDPCSGDAAW&thval=0.0) | -0.07 | Non-Toxin | -0.04 | -0.11 | -0.03 | -2 | 1107.3 |
|  | [IDGNPITGK](https://webs.iiitd.edu.in/raghava/toxinpred/pepsearch_S.php?seq=IDGNPITGK&thval=0.0) | -0.85 | Non-Toxin | -0.1 | -0.56 | 0.24 | 0 | 914.16 |
|  | [DMQGTSMEGPFPSI](https://webs.iiitd.edu.in/raghava/toxinpred/pepsearch_S.php?seq=DMQGTSMEGPFPSI&thval=0.0) | -1.14 | Non-Toxin | -0.05 | -0.41 | -0.04 | -2 | 1496.9 |
|  | [TESPPANCW](https://webs.iiitd.edu.in/raghava/toxinpred/pepsearch_S.php?seq=TESPPANCW&thval=0.0) | -0.16 | Non-Toxin | -0.13 | -0.92 | -0.2 | -1 | 1004.2 |
|  | [SSTNINSIASC](https://webs.iiitd.edu.in/raghava/toxinpred/pepsearch_S.php?seq=SSTNINSIASC&thval=0.0) | -0.77 | Non-Toxin | -0.07 | 0.22 | -0.35 | 0 | 1096.3 |
|  | [INCGGSHVTVDGNEY](https://webs.iiitd.edu.in/raghava/toxinpred/pepsearch_S.php?seq=INCGGSHVTVDGNEY&thval=0.0) | -0.72 | Non-Toxin | -0.07 | -0.39 | -0.15 | -1.5 | 1564.9 |
|  | [EDDTSPQGASR](https://webs.iiitd.edu.in/raghava/toxinpred/pepsearch_S.php?seq=EDDTSPQGASR&thval=0.0) | -0.72 | Non-Toxin | -0.44 | -1.91 | 1.08 | -2 | 1162.3 |
|  | [LISAISVTPNF](https://webs.iiitd.edu.in/raghava/toxinpred/pepsearch_S.php?seq=LISAISVTPNF&thval=0.0) | -1.42 | Non-Toxin | 0.18 | 1.29 | -0.86 | 0 | 1161.5 |
|  | [GIVAASCVVIM](https://webs.iiitd.edu.in/raghava/toxinpred/pepsearch_S.php?seq=GIVAASCVVIM&thval=0.0) | -0.82 | Non-Toxin | 0.34 | 2.58 | -1.01 | 0 | 1062.5 |
|  | [PDGSEIAVK](https://webs.iiitd.edu.in/raghava/toxinpred/pepsearch_S.php?seq=PDGSEIAVK&thval=0.0) | -0.97 | Non-Toxin | -0.12 | -0.36 | 0.61 | -1 | 915.13 |
|  | [INEIGMISA](https://webs.iiitd.edu.in/raghava/toxinpred/pepsearch_S.php?seq=INEIGMISA&thval=0.0) | -0.83 | Non-Toxin | 0.15 | 1 | -0.41 | -1 | 947.25 |
|  | [DEEENTHISTR](https://webs.iiitd.edu.in/raghava/toxinpred/pepsearch_S.php?seq=DEEENTHISTR&thval=0.0) | -0.65 | Non-Toxin | -0.48 | -2.08 | 1.13 | -2.5 | 1330.5 |
|  | [AVGSASPTL](https://webs.iiitd.edu.in/raghava/toxinpred/pepsearch_S.php?seq=AVGSASPTL&thval=0.0) | -0.96 | Non-Toxin | 0.11 | 0.81 | -0.46 | 0 | 802.01 |
|  | [GVDPCSGEGNW](https://webs.iiitd.edu.in/raghava/toxinpred/pepsearch_S.php?seq=GVDPCSGEGNW&thval=0.0) | -0.65 | Non-Toxin | -0.08 | -0.75 | 0.05 | -2 | 1120.3 |
|  | [LESDVVCDCS](https://webs.iiitd.edu.in/raghava/toxinpred/pepsearch_S.php?seq=LESDVVCDCS&thval=0.0) | 0.13 | Toxin | -0.09 | 0.51 | 0.28 | -3 | 1069.3 |
|  | [EGPIPSGISN](https://webs.iiitd.edu.in/raghava/toxinpred/pepsearch_S.php?seq=EGPIPSGISN&thval=0.0) | -1.14 | Non-Toxin | -0.01 | -0.36 | 0.02 | -1 | 970.19 |
|  | [NCSIHGDIPAY](https://webs.iiitd.edu.in/raghava/toxinpred/pepsearch_S.php?seq=NCSIHGDIPAY&thval=0.0) | -0.85 | Non-Toxin | -0.01 | -0.09 | -0.4 | -0.5 | 1189.5 |
|  | [TVGSSGITQC](https://webs.iiitd.edu.in/raghava/toxinpred/pepsearch_S.php?seq=TVGSSGITQC&thval=0.0) | -0.94 | Non-Toxin | 0.01 | 0.39 | -0.43 | 0 | 952.19 |
|  | [EGSVNTVECY](https://webs.iiitd.edu.in/raghava/toxinpred/pepsearch_S.php?seq=EGSVNTVECY&thval=0.0) | 0.03 | Toxin | -0.1 | -0.28 | -0.02 | -2 | 1100.3 |
|  | [TFPCSASNK](https://webs.iiitd.edu.in/raghava/toxinpred/pepsearch_S.php?seq=TFPCSASNK&thval=0.0) | -0.32 | Non-Toxin | -0.18 | -0.47 | -0.07 | 1 | 954.17 |
|  | [HINCGGEETIIK](https://webs.iiitd.edu.in/raghava/toxinpred/pepsearch_S.php?seq=HINCGGEETIIK&thval=0.0) | -0.54 | Non-Toxin | -0.08 | -0.26 | 0.16 | -0.5 | 1313.7 |
|  | [MDDDVNADNY](https://webs.iiitd.edu.in/raghava/toxinpred/pepsearch_S.php?seq=MDDDVNADNY&thval=0.0) | -0.65 | Non-Toxin | -0.31 | -1.44 | 0.68 | -4 | 1171.3 |
|  | [NIEDAAGGPGKPVIK](https://webs.iiitd.edu.in/raghava/toxinpred/pepsearch_S.php?seq=NIEDAAGGPGKPVIK&thval=0.0) | -1.05 | Non-Toxin | -0.09 | -0.39 | 0.41 | 0 | 1465.9 |
|  | [LISAISVTPNF](https://webs.iiitd.edu.in/raghava/toxinpred/pepsearch_S.php?seq=LISAISVTPNF&thval=0.0) | -1.42 | Non-Toxin | 0.18 | 1.29 | -0.86 | 0 | 1161.5 |
|  | [DVGSPSSNR](https://webs.iiitd.edu.in/raghava/toxinpred/pepsearch_S.php?seq=DVGSPSSNR&thval=0.0) | -0.67 | Non-Toxin | -0.36 | -1.3 | 0.62 | 0 | 918.04 |
|  | [TIIVVVVMA](https://webs.iiitd.edu.in/raghava/toxinpred/pepsearch_S.php?seq=TIIVVVVMA&thval=0.0) | -0.84 | Non-Toxin | 0.44 | 3.2 | -1.31 | 0 | 944.38 |
|  | [HDNTAHPAPDS](https://webs.iiitd.edu.in/raghava/toxinpred/pepsearch_S.php?seq=HDNTAHPAPDS&thval=0.0) | -0.51 | Non-Toxin | -0.27 | -1.64 | 0.37 | -1 | 1161.3 |
|  | [GGSDGGPGGGNK](https://webs.iiitd.edu.in/raghava/toxinpred/pepsearch_S.php?seq=GGSDGGPGGGNK&thval=0.0) | -0.74 | Non-Toxin | -0.14 | -1.34 | 0.54 | 0 | 959.12 |
|  | [VNDIIIKPNDR](https://webs.iiitd.edu.in/raghava/toxinpred/pepsearch_S.php?seq=VNDIIIKPNDR&thval=0.0) | -0.87 | Non-Toxin | -0.27 | -0.57 | 0.5 | 0 | 1296.7 |
|  | [HNLLCPETVES](https://webs.iiitd.edu.in/raghava/toxinpred/pepsearch_S.php?seq=HNLLCPETVES&thval=0.0) | -0.94 | Non-Toxin | -0.1 | -0.23 | -0.05 | -1.5 | 1241.5 |
|  | [DDVTAMVPR](https://webs.iiitd.edu.in/raghava/toxinpred/pepsearch_S.php?seq=DDVTAMVPR&thval=0.0) | -1.16 | Non-Toxin | -0.21 | -0.19 | 0.42 | -1 | 1003.3 |
|  | [NTEAHSFPV](https://webs.iiitd.edu.in/raghava/toxinpred/pepsearch_S.php?seq=NTEAHSFPV&thval=0.0) | -0.89 | Non-Toxin | -0.09 | -0.5 | -0.21 | -0.5 | 1001.2 |

Table S3. Prediction of true lectin peptides anticancer score, GRAVY and charge

| ID | No | | Sequence | Score | | Prediction | | GRAVY | | | Charge |
| --- | --- | --- | --- | --- | --- | --- | --- | --- | --- | --- | --- |
| XP_038973335.1 | 1 | | MVYPEPVTIYDEASVIN | 0.33 | | Non-AntiCP | | 0.24 | | | -3 |
|  | 2 | | SSYPSAIPDL | 0.51 | | AntiCP | | -0.03 | | | -1 |
|  | 3 | | TLPSTVVAVE | 0.41 | | Non-AntiCP | | 1.09 | | | -1 |
|  | 4 | | DISANHIGIDVHTIYSVVQ | 0.47 | | AntiCP | | 0.42 | | | -1 |
|  | 5 | | SSQGYGSSPSPTPR | 0.59 | | AntiCP | | -1.4 | | | 1 |
|  | 6 | | EYISEVTIISR | 0.4 | | Non-AntiCP | | 0.24 | | | -1 |
|  | 7 | | HEGWEQCVIHR | 0.15 | | Non-AntiCP | | -1.05 | | | 0 |
|  | 8 | | AGTMGYIAPEYAITGK | 0.47 | | AntiCP | | 0.13 | | | 0 |
|  | 9 | | DTPLPALPPNMPIPMF | 0.55 | | AntiCP | | 0.2 | | | -1 |
|  | 10 | | NVPAPPPNSESCSIV | 0.59 | | AntiCP | | -0.14 | | | -1 |
| XP_008793992.2 | 1 | | NQADSSPSS | 0.68 | | | AntiCP | -1.5 | | -1 | |
|  | 2 | | LSPYPSEIPENSYGGT | 0.54 | | | AntiCP | -0.84 | | -2 | |
|  | 3 | | NVEYNDSSSNHVGIDVHTIF | 0.46 | | | AntiCP | -0.39 | | -2 | |
|  | 4 | | DACVGYDGGAK | 0.58 | | | AntiCP | -0.28 | | -1 | |
|  | 5 | | HWSTSYVVD | 0.44 | | | Non-AntiCP | -0.31 | | -0.5 | |
|  | 6 | | ASVVGAGVVA | 0.2 | | | Non-AntiCP | 2.06 | | 0 | |
|  | 7 | | EQVEMAVAIDS | 0.34 | | | Non-AntiCP | 0.33 | | -3 | |
|  | 8 | | EYISEVTIISR | 0.4 | | | Non-AntiCP | 0.24 | | -1 | |
|  | 9 | | HEEWEQCVVHRVDHDCDPATTV | 0.2 | | | Non-AntiCP | -0.86 | | -3.5 | |
|  | 10 | | DAPLPILSPNMPLPVF | 0.39 | | | Non-AntiCP | 0.67 | | -1 | |
|  | 11 | | LRPPPVDISN | 0.56 | | | AntiCP | -0.46 | | 0 | |
| XP_008795773.2 | 1 | | NGSIPATWASL | 0.76 | | | AntiCP | 0.29 | | 0.00 | |
|  | 2 | | IDGNPISGK | 0.38 | | | Non-AntiCP | -0.57 | | 0.00 | |
|  | 3 | | IDMQGTSMEGPFPPTF | 0.46 | | | AntiCP | -0.28 | | -2.00 | |
|  | 4 | | LAPSSCQEGN | 0.58 | | | AntiCP | -0.60 | | -1.00 | |
|  | 5 | | NMVSSYSSTESNSIAR | 0.43 | | | Non-AntiCP | -0.59 | | 0.00 | |
|  | 6 | | DDHEYEDDPSQMGPSR | 0.36 | | | Non-AntiCP | -2.30 | | -4.50 | |
|  | 7 | | IFDVSIQGQK | 0.5 | | | AntiCP | 0.04 | | 0.00 | |
|  | 8 | | EANGTGRPIIK | 0.34 | | | Non-AntiCP | -0.70 | | 1.00 | |
|  | 9 | | LISAISVTPNF | 0.49 | | | AntiCP | 1.29 | | 0.00 | |
|  | 10 | | TDSKPDIQESK | 0.4 | | | Non-AntiCP | -1.93 | | -1.00 | |
|  | 11 | | FINEIGMISA | 0.5 | | | AntiCP | 1.18 | | -1.00 | |
|  | 12 | | YGCCIEGSQ | 0.47 | | | AntiCP | -0.04 | | -1.00 | |
|  | 13 | | IYEYMENNS | 0.57 | | | AntiCP | -1.22 | | -2.00 | |
|  | 14 | | NEEENTHISTR | 0.78 | | | AntiCP | -2.08 | | -1.50 | |
|  | 15 | | IAGTMGYMAPEYA | 0.47 | | | AntiCP | 0.35 | | -1.00 | |
|  | 16 | | VNTSVNIDQSSK | 0.51 | | | AntiCP | -0.67 | | 0.00 | |
|  | 17 | | NSSSSNISHQAV | 0.48 | | | AntiCP | -0.60 | | 0.50 | |
| XP_008795773.2 | | 1 | SVDPCSGNAGW | 0.36 | | | Non-AntiCP | -0.31 | | -1.00 | |
|  |  | 2 | IDGNPISGK | 0.38 | | | Non-AntiCP | -0.57 | | 0.00 | |
|  |  | 3 | LAPSSCQEGN | 0.58 | | | AntiCP | -0.60 | | -1.00 | |
|  |  | 4 | SSTESNSIAR | 0.49 | | | AntiCP | -0.91 | | 0.00 | |
|  |  | 5 | EDDPSQMGPSR | 0.42 | | | Non-AntiCP | -1.98 | | -2.00 | |
|  |  | 6 | EANGTGRPIIK | 0.34 | | | Non-AntiCP | -0.70 | | 1.00 | |
|  |  | 7 | LISAISVTPNF | 0.49 | | | AntiCP | 1.29 | | 0.00 | |
|  |  | 8 | TDSKPDIQESK | 0.4 | | | Non-AntiCP | -1.93 | | -1.00 | |
|  |  | 9 | INEIGMISA | 0.45 | | | AntiCP | 1.00 | | -1.00 | |
|  |  | 10 | NEEENTHISTR | 0.78 | | | AntiCP | -2.08 | | -1.50 | |
|  |  | 11 | EVISGMSNTNY | 0.56 | | | AntiCP | -0.35 | | -1.00 | |
|  |  | 12 | VNTSVNIDQSSK | 0.51 | | | AntiCP | -0.67 | | 0.00 | |
|  |  | 13 | NSSSSNISHQAV | 0.48 | | | AntiCP | -0.60 | | 0.50 | |
| XP_008792302.2 | | 1 | SVDPCSGDAAW | 0.55 | | | AntiCP | -0.11 | | -2 | |
|  |  | 2 | IDGNPITGK | 0.48 | | | AntiCP | -0.56 | | 0 | |
|  |  | 3 | DMQGTSMEGPFPSI | 0.46 | | | AntiCP | -0.41 | | -2 | |
|  |  | 4 | TESPPANCW | 0.37 | | | Non-AntiCP | -0.92 | | -1 | |
|  |  | 5 | SSTNINSIASC | 0.51 | | | AntiCP | 0.22 | | 0 | |
|  |  | 6 | INCGGSHVTVDGNEY | 0.29 | | | Non-AntiCP | -0.39 | | -1.5 | |
|  |  | 7 | EDDTSPQGASR | 0.34 | | | Non-AntiCP | -1.91 | | -2 | |
|  |  | 8 | LISAISVTPNF | 0.49 | | | AntiCP | 1.29 | | 0 | |
|  |  | 9 | GIVAASCVVIM | 0.16 | | | Non-AntiCP | 2.58 | | 0 | |
|  |  | 10 | PDGSEIAVK | 0.56 | | | AntiCP | -0.36 | | -1 | |
|  |  | 11 | INEIGMISA | 0.45 | | | AntiCP | 1 | | -1 | |
|  |  | 12 | DEEENTHISTR | 0.85 | | | AntiCP | -2.08 | | -2.5 | |
| XP_026655880.2 | | 1 | AVGSASPTL | 0.72 | | | AntiCP | 0.81 | | 0 | |
|  |  | 2 | GVDPCSGEGNW | 0.36 | | | Non-AntiCP | -0.75 | | -2 | |
|  |  | 3 | LESDVVCDCS | 0.24 | | | Non-AntiCP | 0.51 | | -3 | |
|  |  | 4 | EGPIPSGISN | 0.39 | | | Non-AntiCP | -0.36 | | -1 | |
|  |  | 5 | NCSIHGDIPAY | 0.62 | | | AntiCP | -0.09 | | -0.5 | |
|  |  | 6 | TVGSSGITQC | 0.25 | | | Non-AntiCP | 0.39 | | 0 | |
|  |  | 7 | EGSVNTVECY | 0.4 | | | Non-AntiCP | -0.28 | | -2 | |
|  |  | 8 | TFPCSASNK | 0.69 | | | AntiCP | -0.47 | | 1 | |
|  |  | 9 | HINCGGEETIIK | 0.58 | | | AntiCP | -0.26 | | -0.5 | |
|  |  | 10 | MDDDVNADNY | 0.42 | | | Non-AntiCP | -1.44 | | -4 | |
|  |  | 11 | NIEDAAGGPGKPVIK | 0.23 | | | Non-AntiCP | -0.39 | | 0 | |
|  |  | 12 | LISAISVTPNF | 0.49 | | | AntiCP | 1.29 | | 0 | |
|  |  | 13 | DVGSPSSNR | 0.52 | | | AntiCP | -1.3 | | 0 | |
|  |  | 14 | TIIVVVVMA | 0.45 | | | AntiCP | 3.2 | | 0 | |
| XP_038985107.1 | | 1 | HDNTAHPAPDS | 0.64 | AntiCP | | | -1.64 | -1 | | |
|  |  | 2 | GGSDGGPGGGNK | 0.58 | AntiCP | | | -1.34 | 0 | | |
|  |  | 3 | VNDIIIKPNDR | 0.43 | Non-AntiCP | | | -0.57 | 0 | | |
|  |  | 4 | HNLLCPETVES | 0.4 | Non-AntiCP | | | -0.23 | -1.5 | | |
|  |  | 5 | DDVTAMVPR | 0.52 | AntiCP | | | -0.19 | -1 | | |
|  |  | 6 | NTEAHSFPV | 0.43 | Non-AntiCP | | | -0.5 | -0.5 | | |
